# Overexpression of *MoSM1*, encoding for an immunity-inducing protein from *Magnaporthe oryzae*, in rice confers broad-spectrum resistance against fungal and bacterial diseases

**DOI:** 10.1038/srep41037

**Published:** 2017-01-20

**Authors:** Yongbo Hong, Yayun Yang, Huijuan Zhang, Lei Huang, Dayong Li, Fengming Song

**Affiliations:** 1 National Key Laboratory for Rice Biology, Institute of Biotechnology, Zhejiang University, Hangzhou 310058, P. R. China

## Abstract

Potential of *MoSM1*, encoding for a cerato-platanin protein from *Magnaporthe oryzae*, in improvement of rice disease resistance was examined. Transient expression of *MoSM1* in rice leaves initiated hypersensitive response and upregulated expression of defense genes. When transiently expressed in tobacco leaves, MoSM1 targeted to plasma membrane. The *MoSM1*-overexpressing (MoSM1-OE) transgenic rice lines showed an improved resistance, as revealed by the reduced disease severity and decreased *in planta* pathogen growth, against 2 strains belonging to two different races of *M. oryzae*, causing blast disease, and against 2 strains of *Xanthomonas oryzae* pv. *oryzae*, causing bacterial leaf blight disease. However, no alteration in resistance to sheath blight disease was observed in MoSM1-OE lines. The MoSM1-OE plants contained elevated levels of salicylic acid (SA) and jasmonic acid (JA) and constitutively activated the expression of SA and JA signaling-related regulatory and defense genes. Furthermore, the MoSM1-OE plants had no effect on drought and salt stress tolerance and on grain yield. We conclude that MoSM1 confers a broad-spectrum resistance against different pathogens through modulating SA- and JA-mediated signaling pathways without any penalty on abiotic stress tolerance and grain yield, providing a promising potential for application of MoSM1 in improvement of disease resistance in crops.

Plants have evolved to possess two distinct types of innate immune responses, which are referred to as pathogen (microbe)-associated molecular pattern (PAMP)-triggered immunity (PTI) and effector-triggered immunity (ETI)^1^,^2^. PTI, which is triggered by a large number of PAMPs, is the first layer of the innate immune responses^3^,^4^,^5^. On the other hand, ETI, which is triggered by specific interactions between host plant R proteins and pathogen-derived avirulence proteins, is a specialized form of the innate immune responses^6^. In addition to the innate immune responses, plants have also evolved to establish an inducible immune response system, which is activated upon pathogen infection or treatments with natural or synthetic compounds through multiple distinct signal transduction pathways. Systemic acquired resistance (SAR) and induced systemic resistance (ISR) are two well-known forms of the inducible immune responses^7^,^8^,^9^. The induced immune responses often confer durable, broad-spectrum and systemic resistance against a variety of related or unrelated pathogens at the distal tissues from the infection or treatment sites^7^,^10^.

Plant inducible immune response can be triggered by exogenous application of a number of elicitors^10^. During the last decade, extensive studies have identified a large number of microbe-derived compounds including polysaccharides, glycoproteins and secreted proteins that exhibit activity of inducing immune responses in different plant species. Among them, the microbe-derived proteins have been shown to possess great potentials in developing eco-friendly biopesticides or generating novel disease-resistant crop cultivars or germplasms for practical application of disease control. The best example of the microbe-derived proteins with immune-inducing activity in a variety of plants is the Gram-negative phytopathogenic bacteria-secreted harpin proteins, which generally affect virulence in host plants and induce immune responses in nonhost plants^11^,^12^. Numerous studies have demonstrated that harpins, either applied to plants as a main component of formulated biopesticides or ectopically expressed in transgenic crop plants, trigger diverse beneficial effects such as induction of defense responses against diverse biotic (pathogens and insects) and abiotic stresses and enhancement of plant growth^13^. Elicitin and Nep1-like proteins, produced by a number of pathogenic oomycetes such as *Phytophthora* spp., are other examples that have been shown to be capable of triggering inducible immune response in different plants^14^,^15^,^16^,^17^. It was believed that microbe-derived proteinaceous elicitors can offer a promising strategy of durable, broad-spectrum disease control approach. During the recent years, a quite large number of novel proteinaceous elicitors have been isolated from microbes through screening and identification using a combination of molecular, biochemical and proteomic approaches^18^,^19^,^20^,^21^.

The cerato-platanin (CP) family is a newly identified small, secreted and cysteine-rich protein (~150 aa) family from filamentous fungi^22^,^23^,^24^. CP was first identified from the tree pathogen *Ceratocystis platani* (formerly known as *Ceratocystis fimbriata* f. sp. *platani*)^25^ and later was found in genomes of more than 50 fungal species with all kinds of lifestyles including biotrophic and necrotrophic plant pathogens, animal pathogens, mycoparasites, plant-beneficial fungi and saprotrophs^26^. Although CPs are abundantly secreted into the culture filtrate, they remain partially bound in the fungal cell wall^27^,^28^,^29^. Recent studies have shown that CPs function both as fungal virulence factors or plant defense elicitors^23^. For example, BcSpl1 in *Botrytis cinerea* and MoMSP1 in *Magnaporthe oryzae* were found to contribute to the fungal virulence as the knock-out mutants for *BcSpl1* or *MoMSP1* showed reduced virulence in their host plants^30^,^31^,^32^. Meanwhile, it has also been shown that CPs from both pathogenic and beneficial biocontrol fungi can induce locally and systemically some structural, physiological and molecular defense responses, including initiation of hypersensitive response (HR), accumulation of phenolic compounds and phytoalexins, production of reactive oxygen species (ROS), and upregulation of defense-related genes in host and nonhost plants^33^,^34^,^35^,^36^,^37^,^38^,^39^,^40^,^41^,^42^,^43^,^44^,^45^. SM1 and SM2 from *Trichoderma virens*, BcSpl1 from *B. cinerea*, and MoSM1 (also known as MoMSP1) from *M. oryzae* could induce systemic disease resistance in rice, cotton, maize, tobacco, tomato and Arabidopsis against different pathogens^32^,^37^,^38^,^40^,^42^,^45^,^46^,^47^ and the activation of systemic resistance by CP from *C. platani* and BcSpl1 from *B. cinerea* was found to be regulated through stomatal perception, overexpression of salicylic acid (SA)- and ethylene-signaling genes and camalexin biosynthesis^44^,^46^. Further study revealed that the elicitor activity of the *B. cinerea* BcSpl1 resides in a two-peptide motif on the protein surface^29^. It was thus suggested that members of the CP family represent a novel class of proteins with potential PAMP activity as plant defense elicitors used in disease control^22^,^23^,^24^.

In our previous study, we demonstrated that MoSM1 from *M. oryzae*, when transiently expressed in leaves of Arabidopsis plants or stably expressed in transgenic Arabidopsis plants, conferred a broad-spectrum resistance against different pathogens such as *Botrytis cinerea* and *Pseudomonas syringae* pv. *tomato* DC3000, accompanied by accumulation of ROS and up-regulation of defense-related genes^42^. More recently, it was reported that recombinant MoMSP1 triggered cell death and elicited defense responses in rice, leading to potentiation of resistance to *M. oryzae*^32^. In the present study, we explored the potential of MoSM1 in improvement of rice disease resistance. Our results indicate that the *MoSM1*-overexpressing (MoSM1-OE) transgenic rice lines showed an improved resistance against blast disease, caused by *M. oryzae*, and bacterial blight disease, caused by *Xanthomonas oryzae* pv. *oryzae (Xoo*), probably through modulating SA/jasmonic acid (JA) signaling pathways, but did not affect resistance to sheath blight disease, caused by *Rhizoctonia solani*. Furthermore, the MoSM1-OE plants did not affect the drought and salt stress tolerance and some of the important agronomic traits such as grain yield.

## Results

### Transient expression of *MoSM1* in rice elicits HR and activates expression of defense-related genes

**Figure 1:**
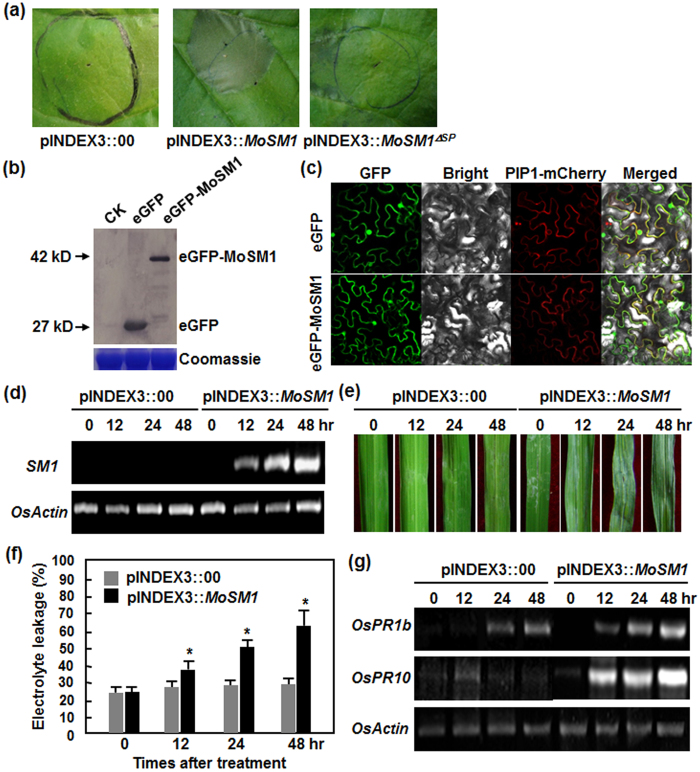
Biochemical characteristics of MoSM1 and activation of rice defense response by transient expression of *MoSM1*. (**a–c**) Biochemical characteristics of MoSM1. (**a**) Requirement of SP for MoSM1 activity. Agrobacteria carrying pINDEX3::MoSM1, pINDEX3::MoSM1^ΔSP^ or pINDEX3::00 were infiltrated into *N. benthamiana* leaves and HR symptom was examined 24 hr later. (**b**) and (**c**) Accumulation and subcellular localization of eGFP-MoSM1 fusion. Agrobacteria carrying pFGC-eGFP-MoSM1 or pFGC-eGFP were co-infiltrated with agrobacteria harboring pCAMBIA1300-AtPIP1;4-mCherry into *N. benthamiana* leaves and leaf samples were collected at 24 hr for Western blotting (**b**) or subcellular localization (**c**). (**d–g**) Activation of rice defense response by MoSM1. Fully expanded leaves of 5-week-old plants were infiltrated with agrobacteria carrying pINDEX3::MoSM1 or pINDEX3::00 construct and induced by spraying with 3 μM dexamethasone 24 hr after agroinfiltration. Leaf samples were collected at indicated time points (hr) and used for analysis of gene expression, measurement of electrolyte leakage and observation of hypersensitive response. (**d**) Expression of *MoSM1* in pINDEX3::MoSM1- and pINDEX3::00-infiltrated leaves. (**e**) HR phenotype caused by transient expression of *MoSM1* in pINDEX3::MoSM1-infiltrated leaves. (**f**) Increased electrolyte leakage caused by transient expression of *MoSM1* in pINDEX3::MoSM1-infiltrated leaves. (**g**) Expression patterns of defense genes in pINDEX3::MoSM1- and pINDEX3::00-infiltrated leaves. Three independent experiments were performed with similar results in (**a**), (**b**), (**c**), (**d**), (**e**) and (**g**). Data presented in (**f**) are the means ± SD from three independent experiments and asterisks indicate statistically significant difference at *p* = 0.05 level between pINDEX3::MoSM1- and pINDEX3::00-infiltrated leaves.

Before the investigation of function of MoSM1 in rice defense, we further examined the biochemical features of MoSM1 such as the role of signal peptide (SP) in activity and subcellular localization *in planta*. Because MoSM1 contains a 21 aa SP at its N-terminal^42^, we first examined whether this SP was necessary for its activity to induce HR. To do this, a SP-deletion vector, pINDEX3::MoSM1^ΔSP^, in which the SP region was deleted, was constructed. At 24 hr after agroinfiltration, transient expression of *MoSM1* in leaves of *N. benthamiana* plants induced a typical HR cell death while transient expression of *MoSM1*^*ΔSP*^ did not (Fig. 1a). We also examined the subcellular localization of MoSM1 *in planta* through generating an eGFP-MoSM1 fusion construct. When transiently expressed in leaves of *N. benthamiana* plants, significant accumulation of eGFP-MoSM1 fusion was detected by Western blotting, which was similar to the accumulation of eGFP alone, at 24 hr after agroinfiltration (Fig. 1b). Microscopic observation revealed that the eGFP-MoSM1 fusion targeted mainly to the plasma membrane, co-localized with a previously reported plasma membrane-targeted Arabidopsis aquaporin AtPIP1;4, whereas the eGFP alone distributed throughout the cells with distribution in nucleus, at 24 hr after agroinfiltration (Fig. 1c). These data suggest that SP in MoSM1 is required for its activity to induce HR and that MoSM1 targets to plasma membrane of cells *in planta*.

We next examined whether transient expression of *MoSM1* in rice could induce defense responses such as HR and expression of defense-related genes. To this end, agrobacteria carrying pINDEX3::MoSM1 and empty pINDEX3::00 were infiltrated into leaves of 5-week-old rice plants and transient expression of *MoSM1* was induced by exogenous application of 3 μM DEX^42^. Transcript of *MoSM1* in pINDEX3::MoSM1-infiltrated leaves was first detected at 12 hr after DEX treatment and the *MoSM1* transcript levels gradually increased over a period of 12–48 hr (Fig. 1d). However, no *MoSM1* transcript was detected in pINDEX3::00-infiltrated leaves (Fig. 1d). Water-soaked symptom initially occurred around the infiltration sites at 12 hr after DEX treatment in pINDEX3::MoSM1-infiltrated leaves and further enlarged and expanded into large area, leading to dried and colorless tissues at 48 hr (Fig. 1e). No obvious water-soaked symptom was observed in pINDEX3::00-infiltrated leaves (Fig. 1e). Similarly, electrolyte leakage in pINDEX3::MoSM1-infiltrated leaves increased significantly by 13%, 26% and 39% at 12, 24 and 48 hr after DEX treatment compared to the level at 0 hr, while the electrolyte leakage in pINDEX3::00-infiltrated leaves had no significant change (Fig. 1f). These results indicate that transient expression of *MoSM1* in rice leaves can induce a typical HR.

We further examined whether transient expression of *MoSM1* activated defense response by comparing the expression patterns of some selected defense-related genes between pINDEX3::MoSM1- and pINDEX3::00-infiltrated leaves after DEX treatment. As shown in Fig. 1g, induced expression of *OsPR1b* and *OsPR10* was detected as early as 12 hr and the expression levels increased further at 24 and 48 hr after DEX treatment in pINDEX3::MoSM1-infiltrated leaves, although relatively lower expression levels of *OsPR1b* and *OsPR10* were also detected in pINDEX3::00-infiltrated leaves. These data indicate that transient expression of *MoSM1* can also activate the expression of defense genes in rice and thus transiently expressed *MoSM1* may function as an elicitor of defense response in rice.

### Generation and characterization of MoSM1-OE transgenic rice lines

**Figure 2:**
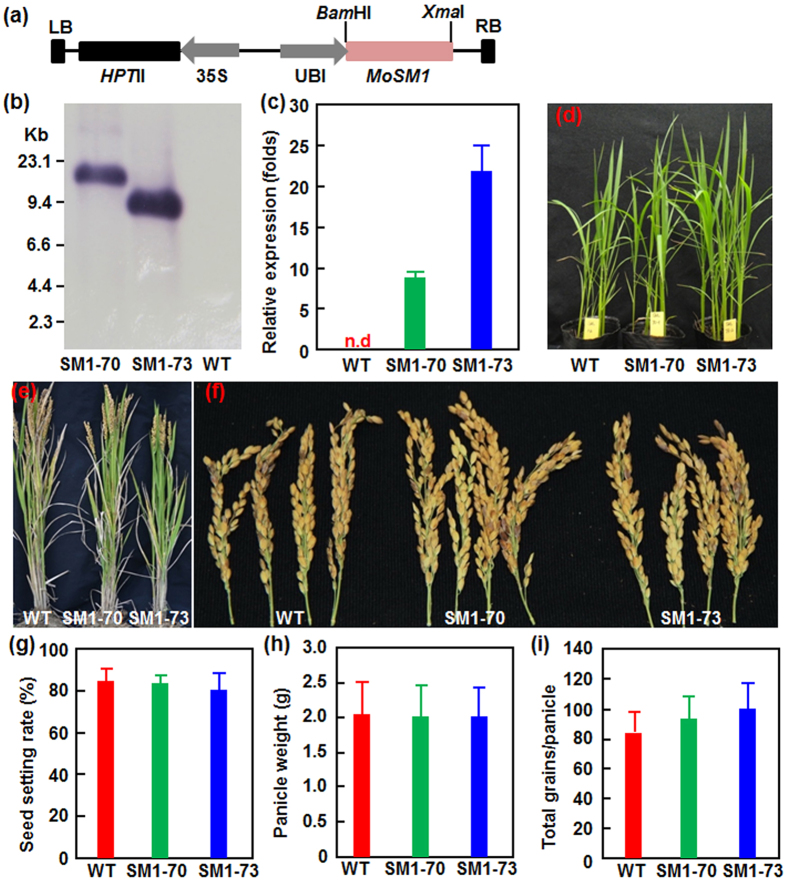
Characterization and growth performance of MoSM1-OE lines. (**a**) Schematic diagram of the overexpression construct for rice transformation. HPT II, Hygromycin phosphotransferase II; LB, left border; RB, right border; Ubi, maize ubiquitin promoter; 35S, CaMV 35S promoter. (**b**) Southern blot analysis of the copy number in two independent transgenic lines. DNA from T_3_ MoSM1-OE and nontransgenic WT plants was digested with *Eco*RI, separated on an agarose gel, transferred to a Hybond-N^+^ nylon membrane and hybridized with a DIG-labelled fragment amplified from the *HptII* gene as a probe. (**c**) Expression levels of *MoSM1* in MoSM1-OE and nontransgenic WT plants. Leaf samples were collected from 8-week-old plants grown in greenhouse and expression of *MoSM1* was analyzed by qRT-PCR. A rice actin gene was used as an internal control and the expression level of *MoSM1* was shown as folds of the *Actin* level. (**d**) and (**e**) Growth phenotype of the MoSM1-OE and nontransgenic WT plants at 3-week-old seedling stage (**d**) and at heading stage (**e**). (**f**) Comparison of the panicles from the MoSM1-OE and nontransgenic WT plants grown in a greenhouse. (**g–i**) Seed setting rates, panicle weight and grains per panicle of the MoSM1-OE and nontransgenic WT plants. Data presented in (**c**) and (**g–i**) are the means ± SD from three independent experiments and no statistically significant difference at *p* = 0.05 level was detected between transgenic and nontransgenic lines.

Transgenic rice lines with overexpression of *MoSM1* (MoSM1-OE) were generated to precisely evaluate the function of MoSM1 in rice immunity. For this purpose, the *MoSM1* full coding sequence including SP was constructed into a binary vector under the control of the maize ubiquitin promoter (Fig. 2a) and transformed into rice cv. Xiushui11. A total 36 independent transgenic lines were obtained through the *Agrobacterium*-mediated transformation method and two independent lines (SM1-70 and SM1-73) with single copy of the *MoSM1* gene, as revealed by Southern blotting (Fig. 2b), were chosen for further studies. qRT-PCR analysis indicated that the expression level of the *MoSM1* gene in plants of the T3 generations of the MoSM1-OE lines SM1-70 and SM1-73 was estimated to be 9 and 22 folds of the level of a rice *Actin* gene, respectively (Fig. 2c). However, no transcript of *MoSM1* was detected in nontransgenic wild type (WT) plants (Fig. 2c). During our study, we did not observe significant changes in morphological and developmental phenotypes in the MoSM1-OE lines SM1-70 and SM1-73 at seedling and adult stages as compared to the WT plants (Fig. 2d and e). Furthermore, we also compared some important agronomic traits such as seed setting rate, panicle weight and grains per panicle of the MoSM1-OE plants with the nontransgenic WT plants grown under greenhouse condition. Overall, the panicles of the MoSM1-OE plants were comparable to those of the nontransgenic WT plants (Fig. 2f). No significant difference in some of key agronomic traits such as seed setting rate, panicle weight and grains per panicle was detected between the MoSM1-OE and nontransgenic WT plants (Fig. 2g–i). Taken together, these data indicate that the MoSM1-OE plants do not have abnormal growth, development and grain production.

### MoSM1-OE plants display an improved resistance against blast disease

**Figure 3:**
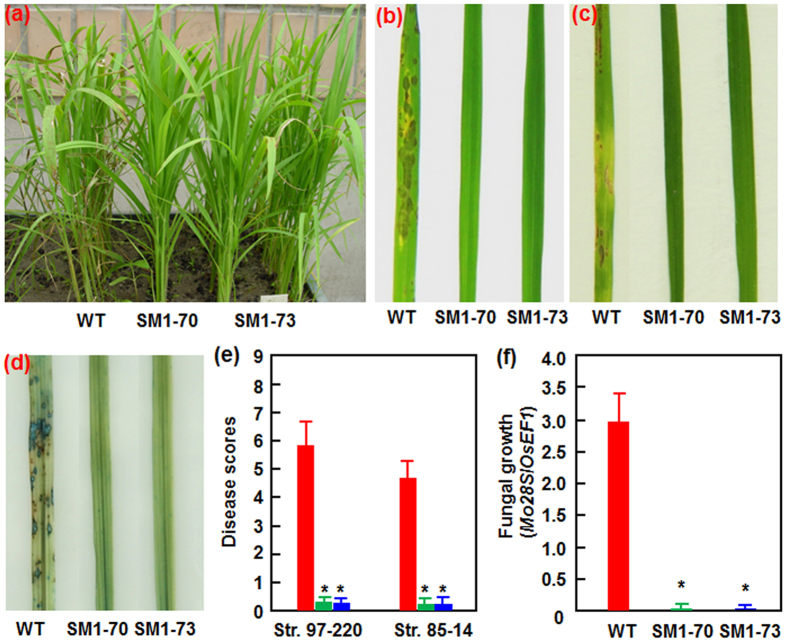
The MoSM1-OE plants display improved resistance against *M. oryzae*. Three-week-old MoSM1-OE and nontransgenic WT plants were inoculated by foliar spraying with spore suspensions (1 × 10^5^ spores/mL) of *M. oryzae* race ZB1 strain 85-14 or race ZE3 strain 97-220. (**a**) Representative disease assays on MoSM1-OE plants inoculated with strain 85-14 in a growth room. (**b**) Disease phenotype on leaves inoculated with strain 85-14 at 7 dpi. (**c**) Disease phenotype on leaves inoculated with strain 97-220 at 7 dpi. (**d**) Trypan blue staining of dead cells in strain 97-220-inoculated leaves of T_3_ transgenic (SM1-70 and SM1-73) and nontransgenic WT plants at 6 dpi. (**e**) Disease scores on leaves inoculated with strains 85-14 and 97-220 at 7 dpi. At least 30 plants in each experiment were evaluated for disease scores using an international nine-scale standard. (**f**) Quantification of fungal growth in strain 97-220-inoculated leaves of the MoSM1-OE and nontransgenic WT plants at 7 dpi. Amounts of *M. oryzae* 28S rDNA and rice *OsEF1* genomic DNA were estimated by qRT-PCR and relative fungal growth were shown as ratios of *Mo28S*/*OsEF1*. Three independent experiments were performed with similar results in (**a**), (**b**) and (**d**). Data presented in (**e**) and (**f**) are the means ± SD from three independent experiments and asterisks indicate statistically significant difference at *p* = 0.05 level between the transgenic and nontransgenic lines.

To explore the function of MoSM1 in rice disease resistance, we first evaluated the resistance level of the MoSM1-OE lines at seedling stage against blast disease, which is the most serious constraint on rice caused by *M. oryzae*. Two *M. oryzae* strains, 85-14 and 97-220, representing races ZB1 and ZE3, respectively, were used to inoculate the two-week-old transgenic and nontransgenic WT seedlings by foliar spraying of spore suspension and disease levels were evaluated by assessing disease scores and measuring *in planta* fungal growth. As shown in Fig. 3a,b and c, the overall blast disease phenotype on the MoSM1-OE lines SM1-70 and SM1-73 was less severe than those in the nontransgenic WT plants after inoculation with strains 85-14 and 97-220. Typical blast lesions were seen on leaves of nontransgenic WT plants, while almost no typical blast lesion was observed on leaves of the MoSM1-OE lines SM1-70 and SM1-73 (Fig. 3a,b and c). Similarly, significant cell death, as revealed by Trypan blue staining, in inoculated leaves of the nontransgenic WT plants was detected, while no remarkable cell death was seen in inoculated leaves of the MoSM1-OE lines SM1-70 and SM1-73 (Fig. 3d). At 7 days post inoculation (dpi) with strains 85-14 and 97-220, the averages of the blast disease scores on the nontransgenic WT plants were 5.8 and 4.7 grades, respectively, while the averages of the blast disease scores on the MoSM1-OE lines SM1-70 and SM1-73 were 0.2–0.3 grades (Fig. 3e), resulting in 4–5 grades lower than those on the WT plants. Further measurement of *in planta* fungal growth, as revealed by analyzing genomic DNA level of the 28S rDNA gene of *M. oryzae*, indicated that the MoSM1-OE lines SM1-70 and SM1-73 supported less growth of *M. oryzae* in inoculated leaves as compared with those in the nontransgenic WT plants (Fig. 3f), leading to a reduction of >110 folds in relative fungal growth. Taken together, these results and observations suggest that the MoSM1-OE lines showed an improved resistance to *M. oryzae*.

### MoSM1-OE plants exhibit an improved resistance against bacterial blight disease

**Figure 4:**
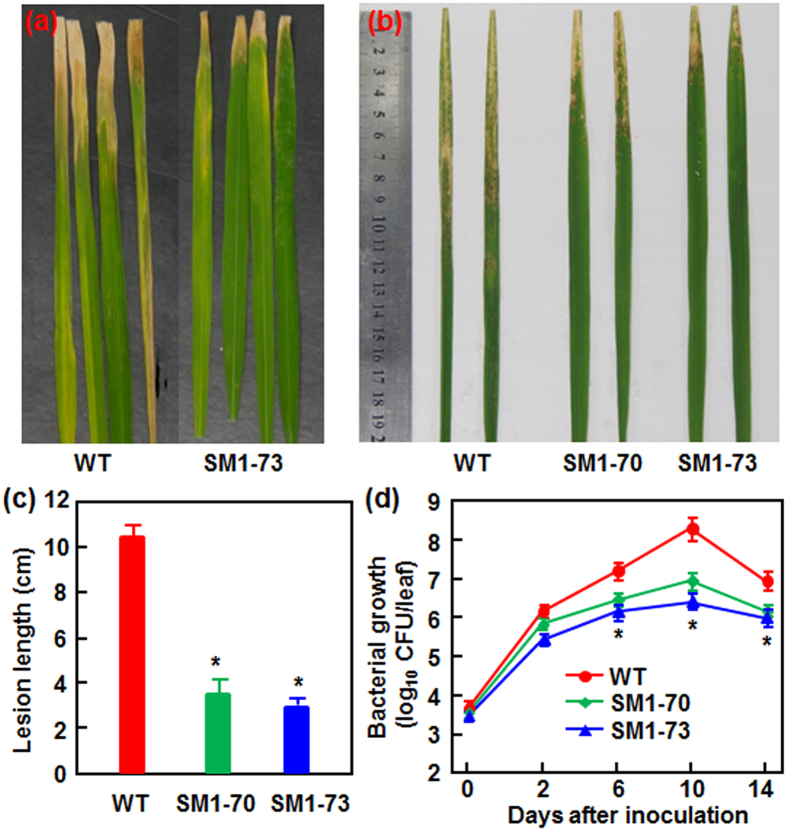
The MoSM1-OE plants display improved resistance against *X. oryzae* pv. *oryzae*. MoSM1-OE and nontransgenic WT plants were inoculated with *X. oryzae* pv. *oryzae* strains PXO96 or Zhe817 using the leaf clipping method at booting stage. (**a**) and (**b**) Disease symptom on the inoculated leaves at 15 dpi. (**c**) Lesion length on the inoculated leaves at 15 dpi. At least 30 plants in each experiment were used for measurement of the lesion lengths. (**d**) Bacterial growth in the PXO96-inoculated leaves of the MoSM1-OE and nontransgenic WT plants. Leaf samples were collected at indicated time points and bacterial growth was determined from three leaves at each time point. Three independent experiments were performed with similar results in (**a**) and (**b**). Data presented in (**c**) and (**d**) are the means ± SD from three independent experiments and asterisks indicate statistically significant difference at *p* = 0.05 level between the transgenic and nontransgenic lines.

We next evaluated the resistance level of the MoSM1-OE lines against bacterial leaf blight disease, which is the most devastating bacterial disease in rice caused by *X. oryzae* pv *oryzae (Xoo*). The MoSM1-OE and nontransgenic WT plants at booting stage were inoculated using the leaf-clipping method with 2 different *Xoo* strains PXO96 and Zhe817 and the disease levels were evaluated by measuring lesion length at 15 dpi and the dynamics of bacterial growth in inoculated leaves. As shown in Fig. 4a and b, the overall bacterial blight disease severity in the MoSM1-OE lines SM1-70 and SM1-73 was lower than that in the nontrangenic WT plants after inoculation with strains PXO96 and Zhe817. At 15 dpi, the average of the lesion length on leaves of nontransgenic WT plants was 10.5 cm, while the averages of the lesion length on leaves of the MoSM1-OE lines SM1-70 and SM1-73 were 3.7 cm and 3.1 cm, respectively (Fig. 4c), leading to 65–70% of reduction in lesion length compared to the nontransgenic WT plants. Similarly, measurement of *in planta* bacterial growth showed that, the growth rates of *Xoo* strains PXO96 in inoculated leaves of the MoSM1-OE lines SM1-70 and SM1-73 were significantly lower than that in the nontransgenic WT plants, resulting in reduction of 10–100 folds at 6, 10 and 14 dpi (Fig. 4d). These results indicate that the MoSM1-OE lines also displayed an improved resistance to *Xoo*.

### MoSM1-OE plants do not alter the resistance against sheath blight disease

**Figure 5:**
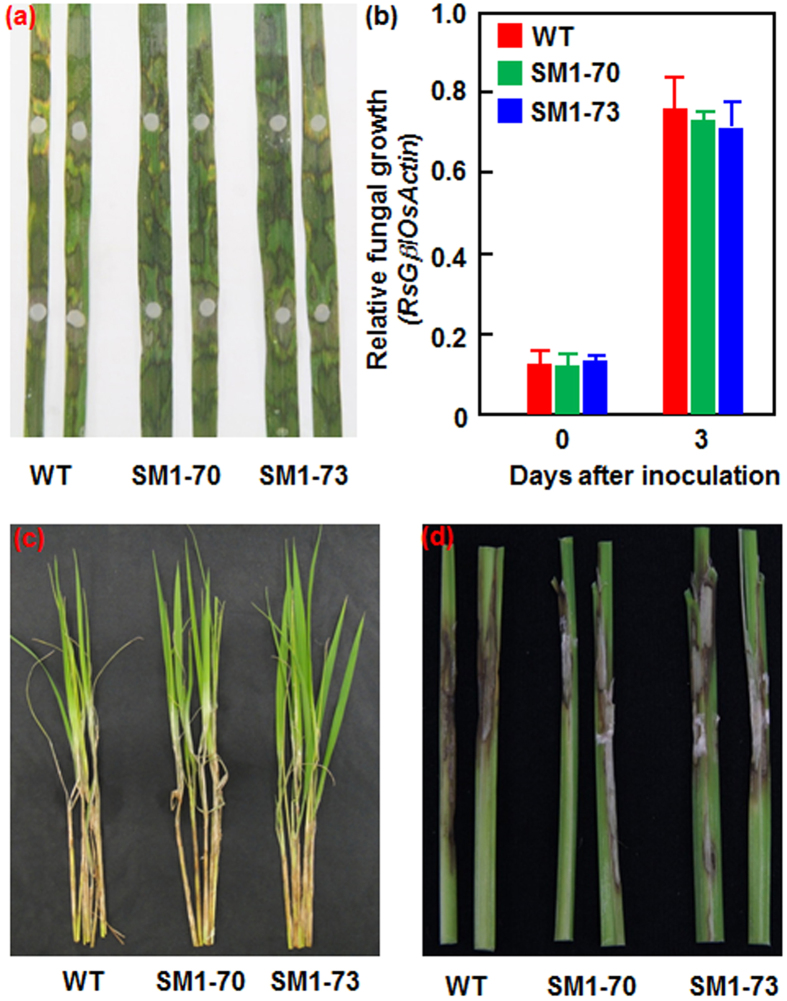
The MoSM1-OE plants show unaltered resistance against *R. solani*. Eight-week-old MoSM1-OE and nontransgenic WT plants were inoculated with *R. solani* strain GD-118 by placing mycelial discs onto detached leaves or attaching mycelial discs on sheath. (**a**) Disease symptom on the inoculated leaves at 3 dpi. (**b**) Quantification of fungal growth in inoculated leaves. Amounts of *R. solani* G protein β-subunit genomic DNA and rice *Actin* genomic DNA were estimated by qRT-PCR and relative fungal growth were shown as ratios of *RsGβ*/*OsActin*. (**c**) and (**d**) Disease symptom on the inoculated sheathes at 15 dpi. At least 20 plants in each experiment were used for assessment of the disease phenotype. Three independent experiments were performed with similar results in (**a**), (**c**) and (**d**). Data presented in (**b**) are the means ± SD from three independent experiments and no statistically significant difference at *p* = 0.05 level was detected between transgenic and nontransgenic lines.

We also evaluated the resistance level of the MoSM1-OE lines against sheath blight disease, another most important fungal disease on rice caused by *R. solani*, which is completely different in infection style from *M. oryzae*. Both *in vitro* detached leaf inoculation assay and *in vivo* sheath inoculation assay were used to evaluate the resistance level of the MoSM1-OE lines to *R. solani*. In the *in vitro* detached leaf inoculation assays, no significant difference in lesion formed on the *R. solani*-inoculated leaves between the MoSM1-OE and nontransgenic WT plants was detected (Fig. 5a). Measurement of *in planta* fungal growth, as revealed by analyzing genomic DNA level of the G-protein β-subunit gene of *R. solani* with a rice *Actin* gene, also indicated no significant difference in fungal growth between the MoSM1-OE and nontransgenic WT plants at 3 dpi (Fig. 5b). In the *in vivo* sheath inoculation assays, lesions on the *R. solani*-inoculated sheath of the MoSM1-OE lines SM1-70 and SM1-73 were comparable in size with those on the sheath of nontransgenic WT plants at 15 dpi (Fig. 5c and d). Collectively, these results suggest that the MoSM1-OE lines did not alter the resistance to *R. solani*.

### MoSM1-OE plants contain elevated SA and JA levels

**Figure 6:**
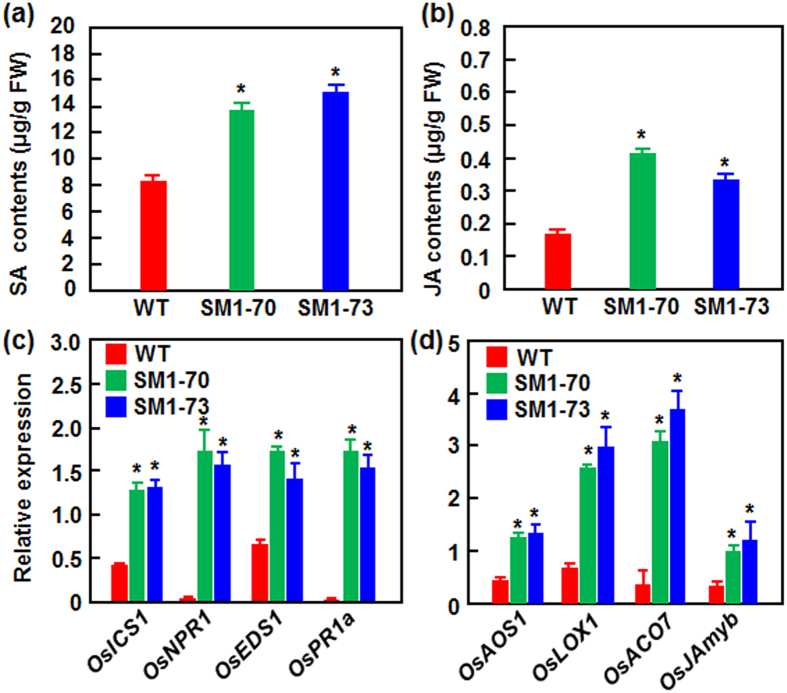
The MoSM1-OE plants contain elevated SA and JA levels and show constitutively activated SA- and JA-mediated signaling pathways. Leaf samples were collected from 4-week-old T_3_ transgenic (SM1-70 and SM1-73) and nontransgenic WT plants grown under normal condition and were used for analyses of hormone contents and gene expression. (**a**) SA content. (**b**) JA content. (**c**) Expression patterns of genes coding for components involved in SA-mediated signaling pathway. (**d**) Expression patterns of genes coding for components involved in JA-mediated signaling pathway. Data presented are the means ± SD from three independent experiments and asterisks indicate statistically significant difference at *p* = 0.05 level between the transgenic and nontransgenic lines. FW, fresh weight.

SA and JA are most important plant hormones that play vital roles in regulating immune responses in rice^48^. To explore the molecular mechanism responsible for the improved resistance in MoSM1-OE lines, we examined whether the MoSM1-OE plants had altered levels of endogenous SA and JA under normal condition. The SA and JA levels in the MoSM1-OE lines SM1-70 and SM1-73 were significantly higher than those in the nontransgenic WT plants, leading to increases of 70–85% for SA and 90–141% for JA (Fig. 6a and b), respectively. These data indicate that the MoSM1-OE plants grown under normal growth condition accumulated elevated SA and JA levels.

### MoSM1-OE plants constitutively activate the SA and JA signaling pathways

The improved resistance and the elevated SA and JA levels let us to examine whether the MoSM1-OE plants constitutively upregulated the expression of SA and JA signaling-related genes. Among them, 4 well-characterized SA pathway genes were selected to compare their expression in the MoSM1-OE and nontransgenic WT plants under normal conditions. As shown in Fig. 6c, the expression levels of *OsICS1, OsEDS1, OsNPR1* and *OsPR1a*, which are involved in the SA biosynthesis, signaling pathway and defense response^49^,^50^, were markedly upregulated in MoSM1-OE plants, showing increases of 2.5, 30, 2.1 and 50 folds, respectively, as compared to the levels in nontransgenic WT plants. Similarly, the expression levels of *OsAOS1, OsLOX1, OsACO7* and *OsJAmyb*, which are involved in JA biosynthesis and signaling pathway^51^,^52^, were also upregulated markedly in MoSM1-OE plants, leading to increases of 2.1, 2.8, 7.8 and 3.1 folds, respectively, as compared to the levels in nontransgenic WT plants (Fig. 6d). These results indicate that the MoSM1-OE plants activated constitutively the SA and JA signaling pathways.

### MoSM1-OE plants do not have penalty on drought and salt stress tolerance

**Figure 7:**
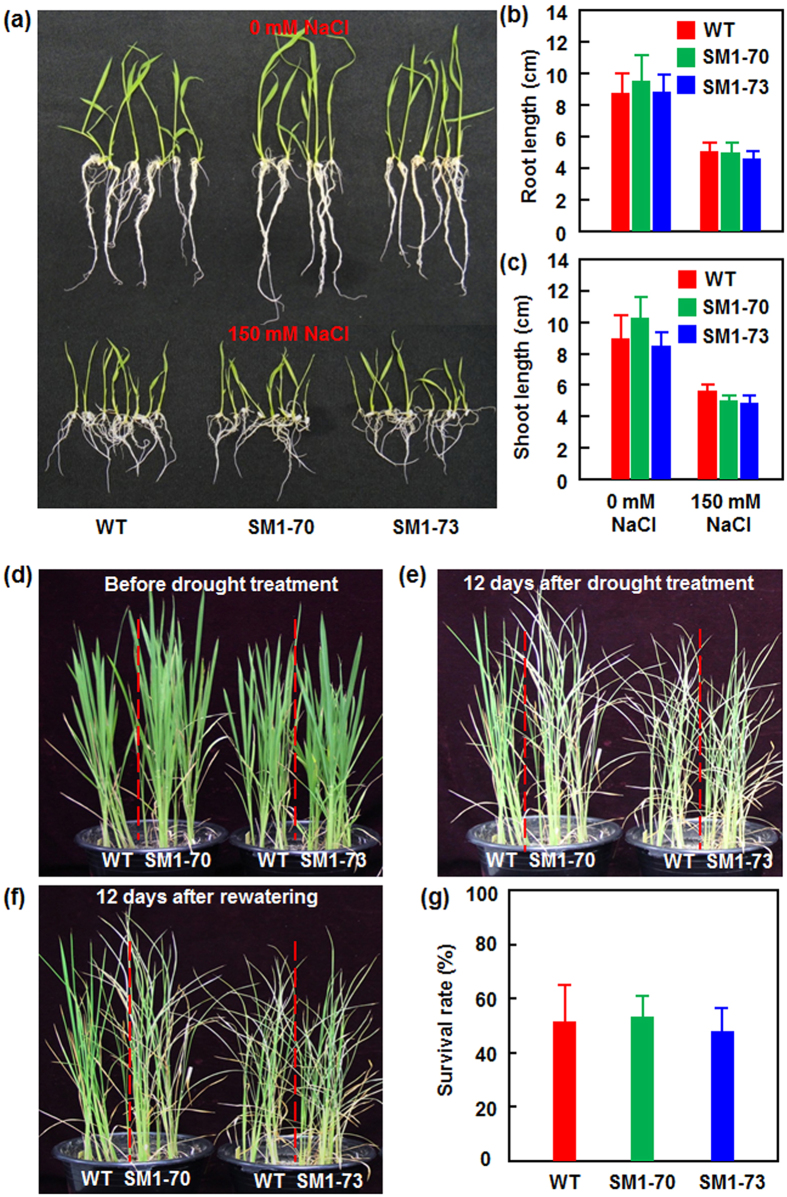
The MoSM1-OE plants show unaltered salt and drought stress tolerance. (**a–c**) Growth performance (**a**), root length (**b**) and shoot length (**c**) of 10-day-old MoSM1-OE and nontransgenic WT seedlings grown on 1/2 MS medium with or without 150 mM NaCl. (**d–f**) Phenotype of 2-week-old MoSM1-OE and nontransgenic WT plants at different stages during the drought stress experiment. (**g**) Survival rates of the MoSM1-OE and nontransgenic WT plants at 12 days after re-watering. Three independent experiments were performed with similar results in (**a**), (**d**), (**e**) and (**f**). Data presented in (**b**), (**c**) and (**g**) are the means ± SD from three independent experiments and no statistically significant difference at *p* = 0.05 level was detected between transgenic and nontransgenic lines.

To examine whether an improved disease resistance in MoSM1-OE lines leads to penalty on other biological processes such as abiotic stress tolerance, we compared the salt and drought tolerance of the MoSM1-OE and nontransgenic WT plants at vegetative stage. In salt stress tolerance assays, no significant difference in seed germination and seedling growth, as revealed by the shoot and root length, was observed between the MoSM1-OE and nontransgenic WT seedlings on 1/2 MS medium with or without supplement of 150 mM NaCl, although the growth of the MoSM1-OE and nontransgenic WT seedlings was markedly inhibited on 1/2 MS medium containing 150 mM NaCl (Fig. 7a,b and c). In drought stress assays, the MoSM1-OE and nontransgenic WT plants showed similar growth status before drought treatment (Fig. 7d). Comparable drought symptom such as leaf rolling and wilting was observed between the MoSM1-OE and nontransgenic WT plants after drought stress treatment by withholding water for 12 days (Fig. 7e) and, no significant difference in growth phenotype (Fig. 7f) and survival rates (Fig. 7g) were observed between the MoSM1-OE and nontransgenic WT plants after re-watering regularly for 12 days. Collectively, these data demonstrate that the MoSM1-OE plants did not have adverse effect on drought and salt stress tolerance.

### MoSM1 levels in transiently expressed leaves and stably expressed transgenic rice

**Figure 8:**
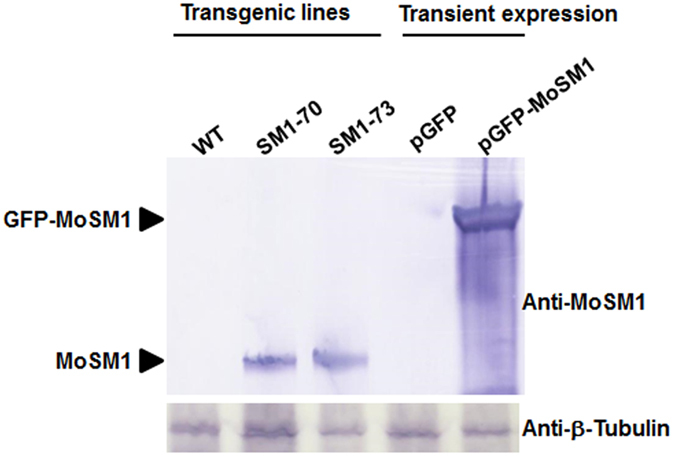
Accumulation of MoSM1 in transiently expressed leaves and stably expressed transgenic rice plants. Protein samples were extracted from transiently expressed leaves or stably expressed leaves of transgenic plants and separated on SDS-PAGE gel. After transferring onto PVDF membrane, MoSM1 was detected using a MoSM1 polyclonal antibody and rice β-tubulin was detected using a β-tubulin monoclonal antibody as an equal-loading control. Three independent experiments were performed with similar results. Cropped blots are displayed and full-length blots are presented in Supplementary Figure S1.

To explain the phenomenon that transient expression of *MoSM1* in rice leaves caused significant cell death but stable expression of *MoSM1* in transgenic rice did not cause visible cell death, we detected the accumulation of MoSM1 in transiently expressed and stably expressed rice leaves. As shown in Fig. 8, a MoSM1 polyclonal antibody, prepared by immunizing rabbits with a peptide CNDLTNGQAGSLGRI, detected the accumulation of MoSM1 in both transiently expressed and stably expressed rice leaves whereas no signal was observed in transiently expressed eGFP leaves or in nontransgenic WT plants. Comparatively, the level of MoSM1 accumulation in transiently expressed rice leaves was relatively higher than those in the MoSM1-OE plants and this was much evident when the MoSM1 accumulation was normalized with the anti-β-tubulin antibody hybridized β-tubulin bands (Fig. 8). Thus, it is likely that the absence of visible cell death in leaves of the MoSM1-OE plants is largely due to a relatively low level of MoSM1, as compared with the amount of MoSM1 and the significant cell death in transiently expressed leaves.

## Discussion

Many members that belong to the cerato-platanin family have been characterized from filamentous fungi including *M. oryzae* and *B. cinerea* and some of them have shown to trigger immunity in a number of plant species^22^,^23^. We and others have shown that, transient or stable expression of *MoSM1* in Arabidopsis or application of recombinant MoMSP1 in rice activated a series of defense responses, which conferred a broad-spectrum resistance against fungal and bacterial pathogens^32^,^42^. The present study further shows that expression of *MoSM1* in transgenic rice confers an improved resistance against blast and bacterial blight diseases, probably through modulating SA/JA signaling pathways, demonstrating that MoSM1 is a promising elicitor that can be used in improvement of rice disease resistance.

No enzymatic activity has been reported to CPs including SM1 of *T. virens*^40^. Although not all reported CPs can induce necrosis in plants^33^,^37^, some CPs including a CP from *C. platani*, MoSM1/MoMSP1 from *M. oryzae* and BcSpl1 from *B. cinerea* have been shown to cause phytotoxic effects on different plants. For instance, when applied at 80 μM^25^, purified *C. platani* CP induced significant necrosis in tobacco leaves and caused several structural and physiological changes, i.e. plasmolysis and accumulation of phenolic compounds^34^,^35^. The *B. cinerea* BcSpl1 was found to produce a faster and stronger necrosis in tobacco leaves than the *C. platani* CP at a lower concentration^31^. Similar to our previous observations that transiently or stably expressed MoSM1 caused typical necrosis in Arabidopsis, tobacco and tomato leaves^42^, we also observed that transiently expressed MoSM1 in rice leaves did cause necrosis symptom and increased leakage of electrolyte (Fig. 1e and f). The difference in phytotoxic effects of the CPs may attribute to the intrinsic differences in CPs, the different plant species used in the assays and/or the methods used for application of the CPs to tested plants^31^. Surprisingly, stably expressed MoSM1 in transgenic rice did not produce any morphological and growth change as compared to the WT plants (Fig. 2d). The difference in appearance of HR-like necrosis in transient expression of *MoSM1* in rice leaves and in stable expression of *MoSM1* in transgenic rice may be caused by the amounts of MoSM1 accumulated in transiently or stably expressed rice plants. We previously found that, when induced with low concentrations of DEX, MoSM1 did not produce toxic effect in MoSM1-OE Arabidopsis plants^42^. Our Western blot assays confirmed that the level of MoSM1 accumulation in transiently expressed rice leaves was relatively higher than those in the MoSM1-OE plants (Fig. 8). This is probably due to the fact of only a single copy of the transgene *MoSM1* in the MoSM1-OE plants vs a quite large number of copies of the *MoSM1* gene in the transiently expressed rice leaves. Thus, unlike the necrosis produced by MoSM1 in transiently expressed rice leaves (Fig. 1e), the amount of MoSM1 in the tissues of the MoSM1-OE plants is not enough to cause observable necrosis in the MoSM1-OE plants. The lack of necrosis in the MoSM1-OE plants features an advantage for the use of these stable transgenic lines in development of novel rice varieties with improved disease resistance as significant necrosis may not be acceptable for practical use.

Most of CPs including MoSM1/MoMSP1 from *M. oryzae* have been shown to induce defense responses related to HR. Several HR characteristic features were observed for some CPs, e.g. the induction of ROS and autofluorescence, appearance of cytoplasm shrinkage and activation of defense gene expression^25^,^31^,^37^,^41^,^44^. The present and our previous studies^42^ showed that MoSM1 can induce visible HR in both Arabidopsis and rice leaves, as verified by the appearance of rapid cell death and the induction of electrolyte leakage, and expression of defense genes (Fig. 1e,f and g). Similar results were also obtained with recombinant MoMSP1, which triggered cell death and induced H_2_O_2_ production in both suspension-cultured cells and rice leaves^32^. On the other hand, it was shown that MoMSP1 is secreted into culture medium *in vitro* and into apoplast *in planta* during infection and that a high level of accumulation of MoMSP1 in rice apoplast is required for activation of defense response^30^,^32^. We observed that transient expression of *MoSM1*^*ΔSP*^, which lacked the SP, in *N. benthamiana* leaves abolished HR (Fig. 1a), which is similar to the previous observation that deletion of SP led to loss of the activity of MoMSP1 to up-regulate expression of defense genes in rice leaves^32^. These data support the hypothesis that MoSM1/MoMSP1, along with other CPs studied so far, indeed function as typical PAMPs that can trigger PTI and that the secretion of MoSM1 into apoplast is critical for its PAMP activity in triggering immune response. Furthermore, our subcellular localization assays revealed that MoSM1 was targeted to the plasma membrane when transiently expressed in *N. benthamiana* leaves (Fig. 1c), which is similar to the observation that *B. cinerea* BcSpl1 associates with the plasma membrane of cells in tomato and tobacco^29^. It was found recently that Arabidopsis BAK1, a critical component of the PAMP signaling complex^53^, plays a role in the induction of necrosis by BcSpl1^31^. Similar to the common knowledge that some well-known PAMPs such as FLS2 for flg22 were found to be recognized by the extracellular regions of the plasma membrane surface-localized pattern recognition receptors^54^, a yet unknown receptor that recognizes MoSM1/MoMSP1 may be localized on the plasma membrane of plant cells. Therefore, it can be speculated that, during infection or when expressed in rice, MoSM1/MoSP1 is secreted through a SP-guided process into apoplast, and is then recognized by a plasma membrane-localized receptor(s), followed by the initiation of HR and PTI. This speculation explains, at least partially, the importance of MoSM1/MoSP1 secretion into apoplast in triggering the immunity^30^,^32^ and the feature of the MoSM1/MoSP1 plasma membrane localization (Fig. 1c). Further identification of MoSM1/MoMSP1 receptor or receptor complex and its associated components will broaden our understanding of the molecular mechanism of MoSM1/MoMSP1, especially the clarification of the earliest events and downstream signaling pathway involved in MoSM1/MoMSP1-induced HR and immunity.

Several PAMPs such as flg22 and lipopolysaccharides have been shown to induce a systemic defense response with the characteristics of SAR. Indeed, a number of CPs from different filamentous fungi, e.g. SM1, SM2 and Epl1 from the biocontrol agents *T. virens* and *T. atroviride*^37^,^40^,^45^,^47^ as well as BcSpl1 from *B. cinerea* and a CP from *C. platani*^29^,^44^, were shown to induce both local and systemic disease resistance in host and nonhost plants against different life style pathogens. In the present study, we found that overexpression of *MoSM1* in transgenic rice plants confers an improved resistance against two strains representing difference races of *M. oryzae* and *Xoo*, as revealed by reduced disease severity and suppressed *in planta* pathogen growth (Figs 3 and 4). Similarly, application of recombinant MoMSP1 at a sub-lethal concentration was also found to potentiate resistance against *M. oryzae*, with no significant cell death on rice leaves^32^. However, the MoSM1-OE plants seem to be more effective to blast disease than to bacterial blight disease, as the blast disease severity and the *in planta* growth of *M. oryzae* were almost completely suppressed (Fig. 3) while the bacterial blight disease severity and the *in planta Xoo* growth were suppressed by ~70% (Fig. 4). In addition, MoSM1 was previously found to induce resistance in Arabidopsis against two necrotrophic fungi (i.e. *B. cinerea* and *A. brassicicola*) and a (hemi)biotrophic bacterial pathogen (i.e. *P. syringae* pv. *tomato* DC3000)^42^. These data indicate that MoSM1, like other CPs from both beneficial and pathogenic fungi^44^,^47^, confers a broad-spectrum resistance against pathogens with different life styles in host and nonhost plants, providing a great potential for application of MoSM1 in genetic improvement of disease resistance in other crop plants. The MoSM1-OE plants did not show a decreased resistance against sheath blight disease, although they did not gain an increased resistance (Fig. 5). This may be due to that the molecular mechanism responsible for defense response against *R. solani* differs from those for defense response against *M. oryzae* and *Xoo*. Similar results were also observed in OsWAK25-OE plants, which show resistance to both *M. oryzae* and *Xoo* but display increased susceptibility to *R. solani*^55^.

SA and JA signalings are effective against both biotrophic and necrotrophic pathogens in rice^48^,^56^. Given that MoSM1 functions as a PAMP, MoSM1 is hypothesized to be recognized by an unknown receptor, resulting in initiation of signal transduction pathway to activate defense response in rice. We found that the MoSM1-OE plants contained elevated levels of both SA and JA, two critical defense signaling hormones^48^, and upregulated expression of SA- and JA-related biosynthesis and signaling genes (Fig. 6), indicating that the MoSM1 plants constitutively activate the SA- and JA-mediated defense signaling pathways. This is similar to the function of MoSM1 in transgenic Arabidopsis plants^42^. Generally, rice plants contain high levels of endogenous SA that are weakly responsive to pathogen infection^57^,^58^; however, SA can act in both of OsNPR1- and OsWRKY45-dependent pathways in rice defense response^56^. The improved resistance in MoSM1-OE plants may be due to the upregulated expression of SA signaling regulatory genes such as *OsNPR1*, whose overexpression in rice leads to constitutive activation of defense response^59^, rather than the elevated level of SA. The involvement of SA signaling in defense response of the MoSM1-OE plants is partially supported by the upregulated expression of *OsPR1s* and *OsPR10* (Figs 1g and 6c), defense genes that are activated by the SA signaling pathway^60^,^61^, by transiently or stably expressed MoSM1 in rice plants. This is in agreement with the observations that a CP from *C. platani* in Arabidopsis and BcSpl1 from *B. cinerea* in tobacco were shown to function through SA signaling^44^,^46^ but differs from the action of MoMSP1 in rice, in which SA suppressed MoMSP1-induced H_2_O_2_ production and cell death in suspension cultured rice cells^32^. Whereas exogenous SA applied at a high concentration (e.g. 5 mM) suppressed MoMSP1-induced cell death and H_2_O_2_ production in suspension cultured cells^32^, MoSM1 in transgenic rice plants promoted endogenous SA level (Fig. 6a). Alternatively, SA is differentially involved in MoSM1/MoMSP1-induced cell death and defense response in rice. On the other hand, manipulation of the JA biosynthesis by overexpression of *OsAOS2*, encoding allene oxide synthase that is a key enzyme involved in JA biosynthetic pathway, increased the endogenous JA level, which led to activation of defense gene expression and enhancement of resistance to *M. oryzae*^62^. MoSM1 activates JA signaling in the MoSM1-OE plants by upregulating the expression of JA biosynthesis and signaling genes (Fig. 6b and d), similar to the observation that JA enhanced MoMSP1-induced cell death in rice^32^. It is therefore likely that JA signaling is involved in MoSM1/MoMSP1-regulated defense response. This is inconsistent with a recent finding that function of the CP from *C. platani* triggers SA- and ethylene (ET)-signaling pathways, but not the JA-signaling pathway, as revealed by the expression pattern of marker genes^44^. Overall, the CP-triggered defense signaling pathways may be complicated and depend on specific CPs and/or the tested plant species.

The effect of CPs on plant growth and development as well as on the abiotic stress tolerance is not known yet. In the present study, we were unable to observe any morphological and developmental changes at seedling and adult stages and grain yield in the MoSM1-OE plants in comparison to the WT plants (Fig. 2), indicating that MoSM1 does not have effect on plant growth and development. On the other hand, we also did not observe distinguishable difference in salt and drought tolerance between the MoSM1-OE and WT plants (Fig. 7), indicating that overexpression of *MoSM1* in transgenic rice did not disturb the abiotic stress tolerance.

## Conclusion

We demonstrate in the present study that MoSM1, a member of cerato-platanin family from *M. oryzae*, can activate defense response in rice and that the MoSM1-OE plants gain an improved broad-spectrum resistance against fungal blast and bacterial blight, two most devastating diseases of rice worldwide. We also show that the enhanced resistance in the MoSM1-OE plants is regulated through a combination of SA- and JA-mediated signaling pathways. Furthermore, the MoSM1-OE plants do not have any deleterious effect on drought and salt tolerance as well as on important agronomic traits. Taken together, our data demonstrate that overexpression of *MoSM1* in rice confers an improved broad-spectrum resistance against blast and bacterial blight diseases without any penalty on abiotic stress tolerance and grain yield, providing a promising potential for application of MoSM1 in improvement of disease resistance in other crop plants. Further identification of putative MoSM1 receptor(s), characterization of the MoSM1-triggered signaling pathway and genome-wide expression profiling of MoSM1-regulated genes will greatly help to elucidate the molecular mechanism by which MoSM1 regulates plant immunity.

## Methods

### Plant materials and growth

Rice (*Oryza sativa* L.) subsp. *indica* cv. Yuanfengzao was used in the transient expression experiments while subsp. *japonica* cv. Xiushui11 was used in transgenic study. Rice and *N. benthamiana* plants were grown in a growth room set at 28 °C, 14 hr light (>3,000 lux)/26 °C, 10 hr dark.

### Transient expression assays

Infiltration suspension of agrobacteria carrying pINDEX3::MoSM1 or empty pINDEX3::00 construct was prepared as described previously^42^. To construct the SP-deletion vector, the fragment of *MoSM1* without SP was amplified using primers MoSM1-ΔSP-F and MoSM1-ΔSP-R (Supplementary Table S1), digested with *Eco*RI/*Sal*I, and then inserted into pINDEX3 vector^63^, yielding pINDEX3::MoSM1^ΔSP^. Agrobacteria carrying pINDEX3::MoSM1, pINDEX3::MoSM1^ΔSP^ or empty pINDEX3::00 were infiltrated into leaves of 8-week-old *N. benthamiana* or 5-week-old rice plants using a 1-mL syringe without a needle. The agroinfiltrated plants were foliar sprayed with 1 μM DEX solution 24 h later to induce the expression of *MoSM1*. Leaf samples were collected at indicated time points after DEX treatment and used for analyses of electrolyte leakage and defense-related gene expression. For measurement of electrolyte leakage, 200 mg leaf samples were put into 5 ml distilled water for 3 hr at room temperature and the initial conductivity was measured using a DDS-IIAT digital conductivity meter. The samples were then boiled for 15 min to disrupt plant tissue, and the total conductivity was measured using the same meter. Percentage of electrolyte leakage was calculated as (initial conductivity before boiling)/(total conductivity after boiling) × 100%. Experiments were repeated three times using at least 6 leaves from 6 individual plants per treatment in each experiment.

### Subcellular localization assays

The *MoSM1* coding region was amplified using primers MoSM1-SL-F and MoSM1-SL-R (Supplementary Table S1), digested with *Bam*HI/*Xba*I, and cloned into pFGC-eGFP, yielding pFGC-eGFP-MoSM1. Similarly, the coding sequence of *AtPIP1;4*, encoding for a previously reported membrane-localized aquaporin^64^, was cloned into pCAMBIA1300-mCherry, yielding pCAMBIA1300-AtPIP1;4-mCherry. The recombinant plasmids pFGC-eGFP-MoSM1, pCAMBIA1300-AtPIP1;4-mCherry and the empty vector pGFP-EGFP were introduced into *A. tumefaciens* strain GV3101 by electroporation using GENE PULSER II Electroporation System (Bio-Rad Laboratories, Hercules, CA, USA). Agrobacteria carrying pFGC-eGFP-MoSM1 or pGFP-EGFP were co-infiltrated separately with agrobacteria harboring pCAMBIA1300-AtPIP1;4-mCherry into leaves of 8-week-old *N. benthamiana* plants using 1-mL needless syringes. The agroinfiltrated plants were allowed to grow in a growth room at 25 °C for 24 hr and agroinfiltrated leaves were collected. GFP/mCherry fluorescence were imaged using a Zeiss LSM710 confocal laser scanning microscope (Zeiss, Oberkochen, Germany).

### Western blotting assays

Samples from transiently expressed leaves of *N. benthamiana* and rice plants or from leaves of the stable transgenic rice plants were ground in 200 μl extraction buffer (50 mM Tris-HCl, pH 7.4, 150 mM NaCl, 1 mM EDTA, 1 mM DTT, 0.1% Triton X-100, 1× protease inhibitor cocktail, and 1 mM PMSF) and then added 100 μl loading buffer. After boiling for 5 min, the extracts were centrifuged at 10000 g for 10 min at 4 °C and 20 μl of the supernatant were separated on a 15% SDS-PAGE gel, followed by transferring onto PVDF membrane via wet electroblotting. Detection of GFP in transiently expressed *N. benthamiana* leaves was performed using a mouse monoclonal GFP antibody (Huaan Company, Hangzhou, China) whereas detection of MoSM1 in transiently expressed rice leaves or stably expressed in transgenic rice plants was carried out using a MoSM1 polyclonal antibody, prepared by immunizing rabbits with peptide CNDLTNGQAGSLGRI-KLH conjugate (GenScript Company, Nanjing, China). In detection of MoSM1 with MoSM1 polyclonal antibody, a β-tubulin monoclonal antibody anti-β-tubulin (Abmart, Shanghai, China), was used to detect endogenous tubulin as an internal equal-loading control.

### Generation and characterization of MoSM1-OE lines

The full coding sequence of *MoSM1* including SP was amplified using primers MoSM1-OE-F and MoSM1-OE-R (Supplementary Table S1), digested with *Bam*HI/*Sma*I, and inserted into the binary vector pCoUm under the control of a maize *ubiquitin* promoter, yielding plasmid pCoUm-Ubi::MoSM1. The resulting construct was introduced into *A. tumefaciens* strain EHA105 and transformed into rice calli of cv. Xiushui11 through the *Agrobacterium-*mediated transformation protocol. The obtained *MoSM1*-overexpressing transgenic lines were screened by planting seeds on 1/2 MS medium containing 50 μg/L hygromycin (Hgr) and lines showing 3:1 (Hgr-resistant: Hgr-susceptible) segregation were selected as candidates of single-copy transgenic lines. Individual lines of T3 generations showing 100% Hgr resistance on selective medium were selected as homozygous lines. To further characterize the single-copy transgenic lines, approximately 50 μg of genomic DNA was digested completely with *Eco*RI, separated by agarose gel electrophoresis, and transferred onto a Hybond-N^+^ nylon membrane (Amersham Biosciences, Little Chalfont, UK). A 589 bp fragment of the *Hpt*II gene was amplified using a pair of primers HptII-Probe-F and HptII-Probe-R (Supplementary Table S1) and labelled with DIG by the random priming method using a DIG High Prime DNA Labeling and Detection kit I (Roche Diagnostics, Shanghai, China). Prehybridization, hybridization and detection were performed according to the manufacturer’s recommendations. Homozygous lines with single-copy of transgene were used for all experiments.

### Disease assays

*M. oryzae* strains 85-14 (race ZB1) and 97-220 (race E3) were grown on CM medium at 25 °C for 10 days and spores were collected to prepare inoculum. Two-week-old seedlings at three leaf stages were inoculated by spraying with 5 × 10^5^ spores/mL spore suspension containing 0.02% Tween-20^65^. The inoculated plants were kept in the dark for 24 hr at 25 °C with 100% relative humidity and then moved to the growth room with same condition. Disease severity was evaluated using a standard international 0–9 scale (0 = resistant and 9 = susceptible) from at least 30 plants for each line at 7 dpi^65^. Fungal growth in inoculated leaves was estimated using qRT-PCR^66^ by analyzing the genomic DNA level of a *M. oryzae* 28S rDNA and relative fungal growth was presented as ratios obtained by comparison of the genomic fungal 28 S rRNA gene levels with a rice *OsEF1* genomic DNA levels. Trypan blue staining was carried out as previously described^67^. Briefly, leaf samples were submerged in trypan blue solution (2.5 mg/mL trypan blue, 25% lactic acid, 23% phenol and 25% glycerol in H_2_O), incubated for 10 min at 70 °C, heated for 2 min and then stained overnight. After de-staining in chloral hydrate solution (25 g in 10 mL of H_2_O) for 3 days, the samples were equilibrated with 70% glycerol before microscopy examination.

*Xoo* strains PXO96 and Zhe817 were grown in NA broth at 28 °C with shaking and the bacteria were collected by centrifugation and diluted in distilled water to OD_600_ = 0.8. Rice plants at booting stage were inoculated using the leaf-clipping method^68^ and the inoculated plants were moved to a greenhouse under environmental conditions at 30 °C in daytime/25 °C in night with natural sunlight. Disease phenotype and lesion length were recorded at 15 dpi. For measurement of bacterial growth in inoculated leaves, leaf samples were collected from three individual plants of each line, surface sterilized in 70% ethanol for 10 sec, homogenized in 200 μL H_2_O and serially diluted to proper concentration. A hundred microliters of the final dilutions were plated on NA agar medium and incubated at 28 °C for 24 hr before counting the colony-forming units^69^.

Two methods were used to evaluate the resistance of the rice lines against sheath blight disease. For whole plant inoculation assays, *R. solani* strain GD-118 was grown in potato dextrose broth on a 28 °C shaker for 3 days and the mycelia were collected by centrifugation. Eight-week-old plants were inoculated by attaching mycelial balls (8 mm in diameter) inside the sheath with aluminium foil^70^ and disease phenotype was investigated at 15 days after inoculation. For detached leaf inoculation assays, *R. solani* strain GD-118 was grown in potato dextrose agar medium and inoculation was performed by placing mycelial agar plugs onto detached leaves. Disease phenotype was examined at 3 days after inoculation. For measurement of fungal growth in inoculated leaves, the mycelial agar plugs inoculated on the detached leaves were removed and genomic DNA was extracted from the leaf tissues. The fungal amount was determined using qRT-PCR by analyzing the genomic DNA level of a *R. solani* G-protein β-subunit gene^71^ and relative fungal growth was presented as ratios of the fungal G-protein β-subunit gene levels with a rice *Actin* gene level.

### Quantification of SA and JA content

Quantification of SA and JA was carried out according to the method described previously^72^. Briefly, 300 mg leaf tissues were ground in liquid nitrogen with mortar and pestle, extracted with 2 mL 80% methanol and kept overnight at −20 °C. After centrifugation at 4 °C for 10 min at 12,000 × g, the supernatant was collected, dried under nitrogen gas and then dissolved in 0.5 mL 2% ammonia solution. The collected extracts were loaded on preconditioned Oasis MAX SPE columns (Waters Corp., Milford, MA) and sequentially washed with ammonia solution (2%) and 2 mL methanol. SA and JA in the column were eluted with 4 mL methanol containing 1% formic acid. The eluent was dried under nitrogen gas and finally dissolved in 0.5 mL 80% methanol. Quantification of SA and JA was performed by a HPLC-Triple quadrupole liquid chromatography-mass spectrometry system (Model 1290/6460, Aglient Technologies, Santa Clara, CA) with stable isotope-labeled SA and JA as standards. The experiments were repeated three times and data were averaged.

### qRT-PCR analyses of gene expression

Total RNA was extracted from frozen leaf samples using TRIzol reagent (Invitrogen, Shanghai, China) and treated with RNase-free DNase (TaKaRa, Dalian, China). First-strand cDNA was synthesized from 1 μg of total RNA using AMV reverse transcriptase (TaKaRa, Dalian, China) according to the manufacturer’s recommendations. The qRT-PCR was performed in a CFX96 real-time PCR system (BioRad, Hercules, CA, USA) using Fast Essential DNA Green Master kit (Roche Diagnostics, Shanghai, China). The rice *Actin* gene was used as an internal control to normalize the data and relative expression levels of genes of interest were calculated using the 2^−ΔΔCT^ method. Gene-specific primers used in qRT-PCR are listed in Supplementary Table S1. Three independent biological samples were performed.

### Drought and salt tolerance assays

For drought tolerance assays, 4-week-old seedlings were grown in the same pot and were subjected to drought stress treatment by withholding watering for 12 days, followed by recovery with normal water supply for another 7 days. Plants with green and healthy young leaves at 12 days after re-watering were regarded as survivals and survival rate was calculated as percentage of the survived plants in total treated plants. At least 10 plants for each line were used in each experiment. For salt tolerance assays, 100 seeds were germinated on 1/2 MS medium supplemented with or without 150 mM NaCl and allowed the seedling growth on the same medium. At 6 days after germination under 28 °C/25 °C (day/night) with a 12 hr photoperiod, shoot height and fresh weight of seedlings of each line were measured. The experiments were repeated three times and data were averaged.

### Evaluation of grain yield

Rice plants were grown in a restricted greenhouse with natural sunlight and agronomic traits were evaluated. Harvested grains were air-dried and store at room temperature for 1 month. Twenty random panicles from each line were chosen for measuring the panicle weight and total grains per panicle according to the conventional methods. Seed setting rate was calculated as percentage of fully filled seeds in total seeds of a single panicle. Experiments were repeated three times and data were averaged.

## Additional Information

**How to cite this article**: Hong, Y. *et al*. Overexpression of *MoSM1*, encoding for an immunity-inducing protein from *Magnaporthe oryzae*, in rice confers broad-spectrum resistance against fungal and bacterial diseases. *Sci. Rep.* **7**, 41037; doi: 10.1038/srep41037 (2017).

**Publisher's note:** Springer Nature remains neutral with regard to jurisdictional claims in published maps and institutional affiliations.

## Supplementary Material

Supplementary Figure S1

Supplementary Table S1
