# Supplementary material for: Overexpression of MoSM1, encoding for an immunity-inducing protein from Magnaporthe oryzae, in rice confers broad-spectrum resistance against fungal and bacterial diseases
Source: Sci Rep. 2017 Jan 20;7:41037. doi: 10.1038/srep41037 (PMC5247740; doi:10.1038/srep41037)

**Overexpression of *MoSM1*, encoding for an immunity-inducing protein from *Magnaporthe oryzae*, in rice confers broad-spectrum resistance against fungal and bacterial diseases**

Yongbo Hong^1^, Yayun Yang^1^, Huijuan Zhang^1^, Lei Huang^1^, Dayong Li^1^, Fengming Song^1, *^

^1^National Key Laboratory for Rice Biology, Institute of Biotechnology, Zhejiang University, Hangzhou 310058, P. R. China

**Supplementary Figure S1**

Full-length Western blotting pictures for Figure 8


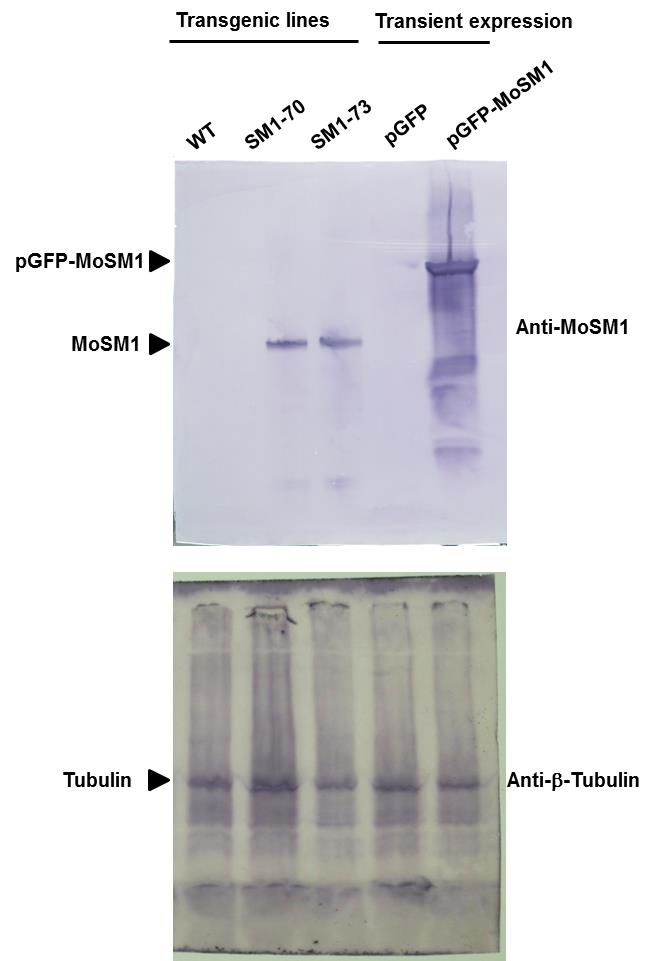

Supplement: Supplementary Figure S1 [file srep41037-s1.doc]
