# Supplementary material for: Overexpression of MoSM1, encoding for an immunity-inducing protein from Magnaporthe oryzae, in rice confers broad-spectrum resistance against fungal and bacterial diseases
Source: Sci Rep. 2017 Jan 20;7:41037. doi: 10.1038/srep41037 (PMC5247740; doi:10.1038/srep41037)
Supplement: Supplementary Table S1 [file srep41037-s2.doc]

**Overexpression of *MoSM1*, encoding for an immunity-inducing protein from *Magnaporthe oryzae*, in rice confers broad-spectrum resistance against fungal and bacterial diseases**

Yongbo Hong^1^, Yayun Yang^1^, Huijuan Zhang^1^, Lei Huang^1^, Dayong Li^1^, Fengming Song^1, *^

^1^National Key Laboratory for Rice Biology, Institute of Biotechnology, Zhejiang University, Hangzhou 310058, P. R. China

**Supplementary Table S1. Primers used in this study**

| Name | Primer sequence (5’-3’) |
| --- | --- |
| ***Plasmid construction*** | |
| MoSM1-ΔSP-F | ATAGAGCTCTCCATCTTCACCCTGGCCGC (*Eco*RI) |
| MoSM1-ΔSP-R | ATAGGATCCCAGGCCGCAGGCGTTGA (*Sal*I) |
| MoSM1-SL-F | GACGGATCCATGCAGTTCTCCAACATCCTC (*Bam*HI) |
| MoSM1-SL-R | GACTCTAGATTACAGGCCGCAGGCGTTGA (*Xba*I) |
| MoSM1-OE-F | GACGGATCCATGCAGTTCTCCAACATCCTC (*Bam*HI) |
| MoSM1-OE-R- | ATACCCGGGTTACAGGCCGCAGGCGTTGA (*Sma*I) |
| ***Southern blotting*** | |
| HptII-Probe-F | ACACAGCCATCGGTCCAGAC |
| HptII-Probe-R | ATCTTAGCCAGACGAGCGGG |
| ***Fungal amount determination*** | |
| 28S rDNA-RT-F | TACGAGAGGAACCGCTCATTCAGATAATTA |
| 28S rDNA-RT-R | TCAGCAGATCGTAACGATAAAGCTACTC |
| Gpbs-RT-F | TCAACGCTGTGCAATTCTTC |
| Gpbs-RT-R | TCCAGTCATCGTATCCTCCA |
| eEF1 alpha-RT-F | CAACCCTGACAAGATTCCCT |
| eEF1 alpha-RT-R | AGTCAAGGTTGGTGGACCTC |
| ***qRT-PCR*** | |
| MoSM1-RT-F | CCAACATCCTCTCCATCTTCAC |
| MoSM1-RT-R | CCAGCCGTACTTGGTGATGAG |
| OsActin-RT-F | AGCTGCGGGTATCCATGAGA |
| OsActin-RT-R | GCAATGCCAGGGAACATAGTG |
| OsPR1a-RT-F | TCGTATGCTATGCTACGTGTTT |
| OsPR1a-RT-F | CACTAAGCAAATACGGCTGACA |
| OsNPR1-RT-F | GCGGCGTCTCCTTGATGTCCTT |
| OsNPR1-RT-F | CGAGTTGTGGGTCCCTTCTTTC |
| OsICS1-RT-F | ACCCTGTTGAACTGTTGGCAT |
| OsICS1-RT-R | TCCGATGAAATAATTGCTCTGG |
| OsEDS1-RT-F | ATAGTTTGCGTGGATTACTTGG |
| OsEDS1-RT-R | GCCTGGTTTGTCTCCCTTTG |
| OsAOS1-RT-F | GGTGAAGAAGGACTACGACCGC |
| OsAOS1-RT-R | CCGAACGAGTTGAAGCAGAGC |
| OsACO7-RT-F | TCGCCACGTTCTACAACCC |
| OsACO7-RT-R | CCTGGAACCTAGCGGTCTTG |
| OsJAmyb-RT-F | TGAAGAGGACTGGGAAGAGCTG |
| OsJAmyb-RT-R | GCTATCTTGGACCATCGGTTGC |
| OsLOX-RT-F | AAACGCTCGCTGGCATCAAC |
| OsLOX-RT-R | ATCGCCTCCTCCACCGTCAT |
